# Human polynucleotide phosphorylase in mitochondrial RNA metabolism

**DOI:** 10.1042/BSR20240504

**Published:** 2025-09-25

**Authors:** Navid Bakshi, Madhuri Kanavalli, Karolina Z. Nowak, Katarzyna J. Bandyra

**Affiliations:** Biological and Chemical Research Centre (CNBCh), Faculty of Chemistry, University of Warsaw, Warsaw 02-089, Poland

**Keywords:** human PNPase, mitochondrial RNA metabolism, PNPT1

## Abstract

Ever since its discovery more than 70 years ago, the enzyme polynucleotide phosphorylase (PNPase) has been the subject of intensive research that has highlighted its key functional roles. The enzyme was first described in 1955 for its ability to synthesise RNA from nucleoside diphosphates. This discovery led to a Nobel Prize in Physiology or Medicine in 1959 for using PNPase to synthesise artificial RNA. However, it soon became evident that the primary function of this enzyme, conserved across diverse species, is 3′-5′ RNA phosphorolysis rather than polymerisation. Remarkably, over 60 years later, it was discovered that PNPase has an even broader range of functions as it was shown to act as a conditional RNA chaperone in bacteria. In humans, PNPase (hPNPase) is located in mitochondria, where it plays a role in mitochondrial RNA (mtRNA) metabolism, thereby regulating mitochondrial function and the overall cell fitness. In this review, we present the current scope of knowledge of hPNPase, including its structure, subcellular localisation, metabolic activity, roles in mtRNA transport, processing and degradation, and its involvement in apoptosis.

## hPNPase is encoded by the *PNPT1* gene and is located in the mitochondria

**Figure 1:**
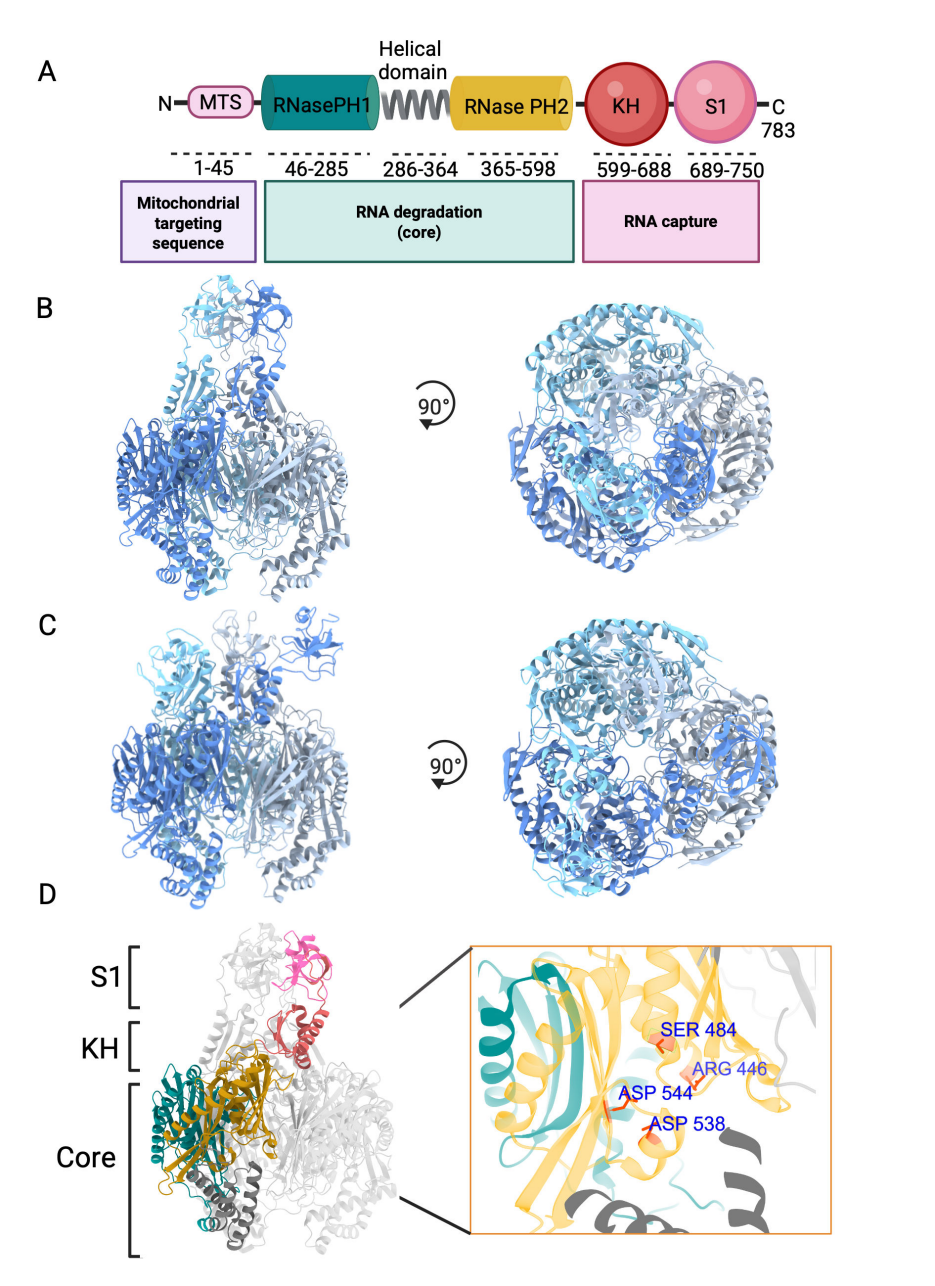
Structure and domain organisation of human PNPase. (**A**) Domain organisation of the hPNPase monomer. The N-terminal mitochondrial targeting sequence (MTS), essential for mitochondrial localisation, is cleaved upon import into the organelle. (**B, C**) Cryo-EM models of the trimeric hPNPase in its closed (B, PDB 9KJT) and open (C, PDB 9KJR) conformations. Side views (left panels) and top views (right panels) are shown, with each protomer coloured in a different shade of blue. (**D**) Domain organisation of a single protomer. Domains are colour-coded as in (**A**). The RNase PH domains, together with the helical domain, form the core region responsible for the catalytic activity of hPNPase. Extending from the core are the KH and S1 domains. The right panel shows a close-up of the active site, with the sidechains of residues critical for catalysis highlighted in red. Created in BioRender. Bandyra, K. (2025) https://BioRender.com/g21u831

Polynucleotide phosphorylase is a highly conserved exoribonuclease found across a wide range of species of bacteria and eukaryotes, which includes plants and complex metazoans. However, it is notably absent in Archaea, trypanosomes and certain unicellular eukaryotes such as *Saccharomyces cerevisiae*, *Schizosaccharomyces pombe* and *Plasmodium falciparum* [1]. The human PNPase locus was identified in 2002 by Leszczyniecka and colleagues as a type I interferon-inducible gene during screening for genes up-regulated in senescence and terminal differentiation [2]. Located in the nucleus, it encodes a 783 amino-acid protein with an N-terminal mitochondrial targeting sequence (MTS) that directs it to the organelle [3,4] (Figure 1A). Within the mitochondria, PNPase predominantly resides in the intermembrane space (IMS) [4]. After translation, the hPNPase polypeptide chain is translocated through the mitochondrial outer membrane via the co-ordinated action of the TOM and TIM23 complexes, the mitochondrial processing peptidase, which removes the MTS releasing the mature protein, and the protease Yme1 that facilitates PNPase translocation to the IMS [5]. In the IMS, PNPase appears to stay associated with a larger membrane complex whose other components are currently unknown [4]. However, a fraction of PNPase localises to the mitochondrial matrix, where it contributes to the assembly of the RNA degradosome complex [6,7].

## hPNPase forms a toroid-shaped trimer

hPNPase belongs to the phosphate-dependent exoribonuclease family of enzymes, which catalyse the phosphate-dependent degradation of RNA, phosphorolysis [1]. Similar to PNPases from other species, the hPNPase protomer consists of five distinct domains: two N-terminal RNase PH domains, 1 and 2, linked by an alpha-helical domain, and two C-terminal RNA-binding domains, KH and S1 [1,8,9] (Figure 1A).

The catalytically active form of PNPase is a trimer, with its active site nestled within the toroidal core formed by six PH domains (Figure 1B and C). A central channel traverses this core, directing RNA substrates to the active site, where key residues – Arg446, Ser484, Asp538 and Asp544 – are essential for catalytic activity [8,10–14] (Figure 1). Flexibly linked to this core are the S1 and KH RNA-binding domains, which play a critical role in capturing and orienting substrates by threading the single-stranded 3′ end of RNA towards the active site. In hPNPase, KH domains adopt conformations distinct from those observed in bacterial homologues. These domains not only extend the central channel and participate in RNA binding but also contribute to the hPNPase trimer stability through interactions with each other and with the N-terminal RNase PH1 domains of the adjacent protomers [8,11,15]. The characteristic for KH domains GXXG motif [16] is also present in hPNPase and plays a vital role in RNA binding. Substitution of the first glycine in this motif with aspartate markedly impairs RNA binding and, consequently, hPNPase catalytic activity [8,16,17]. The KH pore and the central channel entrance exhibit a narrowest diameter of approximately 4 Å, which is insufficient to accommodate single-stranded RNA. This suggests that the KH domains function as an allosterically regulated gate, capable of modulating pore diameter in response to substrate binding [8].

A recent electron cryo-microscopy study of full-length apo-hPNPase identified two distinct conformational states of the enzyme: open and closed, and revealed a substantial conformational shift of the S1 domains between these states [15]. In the open conformation, the S1 domains are positioned apart and remain near the RNase PH2 domains of adjacent protomers, whereas in the closed conformation, they converge and interact with one another. The authors proposed that this conformational transition mimics RNA binding, with the closed state representing the enzyme in a substrate engaged form [15]. Unlike the KH domain, the S1 domain is not essential for RNA binding in hPNPase, as demonstrated by electrophoretic mobility shift assays comparing the wildtype protein with the ΔS1 hPNPase variant [8].

Conformational switching of the S1 domains is crucial for hPNPase activity, both in its isolated form and as part of the mitochondrial degradosome. Disease-associated mutations such as P467S and G499R, although located distantly from the RNA-binding site, were found to favour the open conformation of the enzyme. These mutations lie within the loop region of the RNase PH2 domain in proximity to the S1 domain and are thought to restrict S1 domain flexibility, thereby impairing RNA degradation activity [15]. Interestingly, another disease-linked mutation, D713Y, located directly within the S1 domain, does not affect catalytic activity against single stranded or structured RNA. However, it significantly disrupts the formation of the mtRNA degradosome complex, highlighting the S1 domain’s critical role in mediating protein–protein interactions within this assembly [15].

Other disease-associated hPNPase variants with single amino acid substitutions, Q387R and E475G, disrupt the protomer-protomer interface within the trimer, resulting in the formation of dimers rather than the catalytically active trimers. These dimers exhibit markedly reduced catalytic activity and impaired RNA-binding capacity [10]. In the dimeric state, the KH and S1 domains become inaccessible for RNA interactions, rendering hPNPase functionally deficient in mtRNA metabolism. Structural studies of truncated mutant proteins have shown that in the dimeric form, the KH domains no longer engage in the interdomain interactions seen in the trimeric form. Instead, they interact with the second RNase PH domain of the other protomer, spatially separating the GXXG motifs critical for RNA binding in the KH pore of the trimer. Furthermore, catalytically essential residues are exposed on the surface of the dimer, compromising enzymatic function [10]. SAXS analyses revealed that in these pathogenic dimers, the S1 domains remain flexible and alternate between open (extended) and closed (sequestered) conformations on the protein surface [10].

## hPNPase is 3′-5′exoribonuclease

PNPases are processive enzymes that catalyse the phosphorolysis of RNA from its 3′ end, using inorganic phosphate to cleave the phosphodiester bonds. This reaction requires magnesium ions and releases nucleoside diphosphates, distinguishing it from hydrolytic degradation, which produces nucleoside monophosphates. In addition to RNA degradation, PNPases can catalyse a reverse reaction, leading to RNA polymerisation. In the presence of nucleoside diphosphates, they can add random, template-independent tails to the RNA 3′ end. However, this activity has not yet been demonstrated to occur *in vivo* for hPNPase.

Comparative studies have revealed that while hPNPase shares core mechanistic features with its bacterial and plant chloroplast counterparts, it also exhibits distinct functional properties. In terms of substrate preference, hPNPase differs from its bacterial and plant homologues by lacking a strong preference for polyA RNA, either for degradation or binding [13]. However, in specific contexts, it might show increased activity towards polyadenylated transcripts [18]. hPNPase demonstrates high affinity for polyG and polyC RNA sequences and, similarly to *Escherichia coli* PNPase, is capable of degrading certain structured RNAs under optimal *in vitro* conditions [13].

The enzyme’s activity is also influenced by phosphate concentration, which varies in optimal range between species. While *E. coli* and chloroplast PNPases are most active at phosphate concentrations exceeding 10 nM, hPNPase *in vitro* demonstrates optimal activity at much lower levels, 0.1 mM, and is inhibited at higher concentrations [12,13,19]. Given the relatively high phosphate concentrations reported in mitochondria [20,21], this may have important implications for the regulation of hPNPase activity *in vivo*.

## hPNPase forms a mitochondrial degradosome together with helicase SUV3

PNPase does not appear to couple the energy of phosphorolysis with substrate translocation, as it can stall on structured RNA elements. However, its RNA degradation efficiency increases significantly through interactions with partner proteins in enzymatic complexes known as RNA degradosomes. In bacteria, PNPase commonly associates with DEAD-box RNA helicases such as RhlB (reviewed in [22]). In human mitochondria, hPNPase forms a likely direct interaction with the ATP-dependent RNA helicase SUPV3L1 (SUV3), a core component of the mitochondrial degradosome, to facilitate efficient RNA turnover [6,15,23–25]. A catalytically inactive SUV3 mutant, bearing a deletion of residues 510–514 that disrupts its interaction with hPNPase, has been shown to impair mtRNA degradation. The functional degradosome complex consists of a SUV3 dimer and an hPNPase trimer and exhibits an ATP-dependent degradation activity, also towards double-stranded RNA (dsRNA), particularly those with 3′ overhangs [6,24]. Notably, SUV3 enhances hPNPase degradative efficiency even under conditions of elevated phosphate concentration [24,26], emphasising the importance of this complex in maintaining effective mtRNA decay within human mitochondria.

The interaction between hPNPase and SUV3 predominantly occurs in distinct mitochondrial subcompartments known as D-foci*,* which colocalise with both mtRNA granules and nucleoids. These foci are thought to be essential for efficient RNA decay within mitochondria, potentially providing a specialised microenvironment distinct from the broader mitochondrial matrix, offering more favourable conditions for the optimal enzyme activity. Interestingly, the degradosome remains associated with mitochondrial RNA and DNA, and its localisation is maintained even when the enzymatic activity of its components is disrupted [6,27].

The mitochondrial degradosome works in collaboration with several other proteins to ensure the efficient and precise degradation of mtRNA. The LRPPRC/SLIRP complex has been shown to inhibit degradosome activity by binding to a subset of mRNAs within their coding regions, thereby protecting them from degradation [28]. Since LRPPRC/SLIRP also facilitates the recruitment of mitochondrial mRNAs to modulate their translation, this protective effect might be mediated by the presence of actively translating ribosomes [29]. GRSF1 protein, which facilitates the unwinding of G-quadruplex structures, has been demonstrated to assist the degradosome in the degradation of G-rich RNAs [30]. Given that mitochondrial light strand transcripts are G-rich, the GRSF1-mediated resolution of G-quadruplexes significantly affects the degradosome-dependent decay of non-coding RNAs. The FASTK protein has also been implicated in degradosome regulation, specifically in modulating its activity towards the 3′ untranslated region of ND6 mRNA, thereby ensuring proper transcript maturation [31]. Recently, hPNPase was found to be recruited by the mitochondrial protein C1QBP to degrade RNAs marked with 5-methylcytosine, a process that affects the abundance of mitochondrial dsRNA (mt-dsRNA) [32].

While hPNPase is primarily known as a 3′ to 5′ exoribonuclease, it can also function as an RNA polymerase, highlighting that RNA degradation is not its sole activity. Notably, bacterial PNPase has recently been shown to act as an RNA chaperone for small noncoding RNAs (sRNAs) [33]. In *E. coli*, PNPase forms a carrier complex with the RNA chaperone Hfq, stabilising sRNAs and enhancing the efficiency of post-transcriptional regulation. This multifunctionality raises the possibility that hPNPase may also perform roles beyond RNA degradation, potentially participating in other aspects of mtRNA metabolism.

## hPNPase depletion results in perturbed RNA metabolism

The effects of hPNPase silencing on mtRNA metabolism have been reported with some inconsistency, likely due to variations in the degree of *PNPT1* gene silencing, the specific RNA species or processes examined (e.g. steady-state levels, truncated transcripts, mirror RNAs, or degradation intermediates) and differences in data analysis methodologies. While some studies suggest that hPNPase has minimal impact on mtRNA steady-state levels [4,34], implying a limited involvement in RNA degradation, a substantial body of evidence points to its critical, albeit variable, role in mtRNA metabolism.

hPNPase depletion has been shown to lead to the accumulation of RNA decay intermediates from both the heavy (H) and light (L) mtDNA transcripts, prolonged RNA half-lives and impaired cell growth, ultimately leading to cell division arrest [6]. Moreover, it also results in significant alterations in RNA steady-state levels. For instance, mirror RNAs, antisense transcripts generated during full transcription of both mitochondrial DNA strands and certain non-coding RNAs accumulate to high levels, suggesting that antisense transcripts are the primary degradosome substrates [6,30]. Conversely, the influence on mature mitochondrial mRNAs varies significantly across individual transcripts, with some showing substantial reductions [6]. Other studies reported a near-complete inhibition of mtRNA degradation upon hPNPase silencing, along with polyA tail elongation on several transcripts, implicating the degradosome role in mRNA turnover [28]. Additionally, the silencing of hPNPase alters RNA processing and polyadenylation patterns [35,36], although no direct enzymatic involvement of hPNPase in 3′ ends extension has been demonstrated to date. Despite discrepancies across studies, the consensus remains that hPNPase is essential for maintaining mtRNA homeostasis.

Functionally, hPNPase deficiency leads to significant disruptions in mitochondrial physiology. Its silencing has been shown to inhibit interferon-β-induced cell growth arrest. This, combined with the observation that IFN-β stimulation leads to increased hPNPase expression, underscores the critical role of hPNPase in mediating interferon responses [37]. Moreover, hPNPase deficiency also affects the mitochondrial respiratory chain and morphology, resulting in fragmentation and fission of mitochondria, impaired mitochondrial membrane electrochemical potential, reduced ATP levels and lactate accumulation, ultimately leading to diminished cell growth without a corresponding increase in cell death [4]. As a critical regulator of mitochondrial homeostasis, hPNPase is indispensable for development, with studies showing that constitutive knockout of the *PNPT1* gene in mice results in embryonic lethality [4,38].

## hPNPase prevents the accumulation and release of mt-dsRNAs

A key function of the mitochondrial degradosome is the removal of antisense transcripts that could otherwise lead to accumulation of mt-dsRNA. Owing to the bidirectional transcription of the mitochondrial genome, overlapping H- and L-strand RNA transcripts can form extended double-stranded molecules. These RNAs, containing coding sequences for essential mitochondrial proteins and tRNAs, must be further processed to release functional single-stranded forms. Both hPNPase and SUV3 play a critical role in preventing mt-dsRNA accumulation by ensuring almost complete degradation of the L-strand transcript [6]. Loss of hPNPase leads to leakage of mt-dsRNA into the cytoplasm, where it is recognised by specialised receptors, triggering a type I interferon response [39]. This underscores hPNPase’s essential role in maintaining mtRNA integrity and preventing aberrant activation of innate immune pathways.

Mounting evidence links mt-dsRNA with various inflammatory conditions, where reduced hPNPase activity is a common contributing factor. Patients with diminished hPNPase function often exhibit accumulation of mt-dsRNA and elevated serum interferon levels, highlighting the enzyme’s role in RNA homeostasis and immune regulation [39]. Ethanol exposure has been shown to suppress hPNPase expression in hepatocytes, resulting in the accumulation and cytosolic escape of mt-dsRNA. This in turn triggers the production of interleukin-1β, and subsequently interleukin-17A, contributing to the progression of alcohol-associated liver disease [40]. Similarly, reduced hPNPase levels in renal tubular cells promote mt-dsRNA release, inducing translational arrest and tubular atrophy, a hallmark of chronic kidney disease [41]. mt-dsRNA release has also been observed in osteoarthritis and Huntington’s disease [42,43] and has recently been proposed to act as a mitochondria-derived damage-associated molecular pattern, signalling mitochondrial stress through a regulated export process in which hPNPase plays a pivotal role. Notably, these immunostimulatory RNAs have been detected in certain lung cancer cell lines, highlighting their potential significance in cancer biology [44].

Recent studies have revealed that hPNPase-dependent release of mt-dsRNA is a general feature of cellular senescence across diverse cell types. The cytosolic presence of mt-dsRNA is a key driver of the senescence-associated secretory phenotype, characterised by the secretion of pro-inflammatory factors. This relocation of mt-dsRNA is attributed to decreased hPNPase levels in senescent cells [45]. Since senescent cells directly contribute to the progression of numerous ageing-related diseases and accumulate in aged tissues, these findings provide critical insights into the molecular mechanisms of ageing and its associated disorders.

## hPNPase participates in different aspects of mtRNA metabolism

The mitochondrial degradosome plays a crucial role in preventing the accumulation of R-loops – DNA-RNA hybrids with displaced single-stranded DNA that can arise during transcription or replication. If unresolved, R-loops can interfere with both these processes, leading to genomic instability. The helicase activity of SUV3, coupled with exonuclease activity of hPNPase, facilitates R-loop resolution [34]. Loss of degradosome function results in co-transcriptional R-loop accumulation, particularly at known R-loop hotspots, leading to mitochondrial DNA (mtDNA) instability and depletion [34]. This function likely underlies the observed co-localisation of hPNPase and SUV3 with mitochondrial nucleoids [46,47]. hPNPase has also been directly implicated in the maintenance of mtDNA. In immortalised mouse embryonic fibroblasts, complete loss of the mitochondrial genome was observed in the absence of hPNPase [48]. Similarly, in HeLa cells, hPNPase knockdown led to a significant reduction in mtDNA levels, down to 15% within four days [28]. However, conflicting evidence exists, as another study reported no noticeable changes in mtDNA levels following hPNPase silencing [6], highlighting potential context-dependent variability.

Interestingly, expressing hPNPase in *E. coli* in place of its bacterial homologue leads to R-loop accumulation and RNA stabilisation [49]. Moreover, hPNPase expression in *E. coli* induces oxidative stress and activates the SOS response, mirroring the effects observed in human cells upon hPNPase overexpression. In HeLa cells, elevated hPNPase levels are associated with reactive oxygen species (ROS) production, activation of the NF-κB pathway and up-regulation of proinflammatory cytokines [50]. This pro-oxidative role of hPNPase might result from aberrant mtRNA processing and degradation at elevated protein levels, potentially impairing respiratory activity. Paradoxically, hPNPase is also implicated in the removal of oxidised RNAs, highlighting the complexity of its role in oxidative stress.

Oxidative stress, characterised by the excessive production of ROS, damages proteins, lipids and nucleic acids (reviewed in [51]). Guanine is particularly susceptible, and its oxidation leads to the formation of 8-oxo-7,8-dihydroguanine (8-oxoGua), which can be incorporated into both DNA and RNA, where it mispairs with cytosine or adenine. While cells possess DNA repair mechanisms to address oxidative damage in DNA, no analogous RNA repair pathways have been identified to date. Consequently, the accumulation of oxidised RNA represents a significant threat to cellular homeostasis. hPNPase might help mitigate this threat by contributing to the removal of oxidised RNAs, as it demonstrates a high affinity for RNA molecules containing 8-oxoGua [52]. This protective role is further supported by functional studies showing that hPNPase overexpression enhances the viability of HeLa cells exposed to hydrogen peroxide, a common ROS, whereas its depletion has the opposite effect [53]. These observations suggest a dual, context-dependent role of hPNPase in oxidative stress. The variability in reported outcomes could be influenced by factors such as differences in *PNPT1* expression levels, cell types used, or the presence of some other regulatory factors. Nevertheless, the precise mechanisms by which hPNPase recognises and processes oxidised RNAs remain to be fully elucidated.

## hPNPase mutations are associated with disease

Mutations in *PNPT1,* the gene encoding hPNPase, have been associated with a variety of diseases (Table 1), including OXPHOS deficiency [56,58], hereditary hearing loss [67], Leigh syndrome [55], autoinflammatory disorders like Aicardi-Goutieres syndrome [59], and delayed myelination [54]. Several additional cases with biallelic *PNPT1* variants have been reported, often presenting with considerable clinical heterogeneity [62]. These mutations frequently result in defects in mtRNA processing or impaired RNA transport to mitochondria [10,54,55,58,62]. In some cases, the mutations disrupt the formation of hPNPase trimers, leading to a loss of enzymatic function [10,58,67]. More recent studies have identified mutations that restrict the mobility of the S1 domain, thereby compromising RNA processing and downstream cellular functions. Other mutations have been found to affect the assembly of the mitochondrial degradosome, reducing its stability and diminishing the efficiency of RNA degradation within the mitochondrial matrix [15].

**Table 1:** Summary of hPNPase mutations: their localisation, functional outcomes and associated phenotypes. The sidebars indicate the specific hPNPase domain where each mutation is localised

hPNPase domain	Mutation	Effect	Clinical implication	Reference	hPNPase domain
RPH1	S70P	Reduced protein level	Truncal hypotonia, nystagmus, sensorineural deafness, hypertonia of the lower limbs, and delayed myelination	[39]	RPH1
	G76D	Defective trimer formation	Delayed myelination	[54]	
	D135G	Inability to form trimers		[13]	
	R136H/P140L	Active site disruption	Leigh syndrome	[55]	
	R192X (X = Nonsense mutation)	Defective trimer formation	Delayed myelination	[54]	
	Q254K	Impaired mitochondrial translation and impaired hPNPase assembly	OXPHOS deficiency and decreased hPNPase levels in blood cells	[56]	
HD	K345E	Altered secondary and tertiary structure	Combined oxidative phosphorylation deficiency 13 (COXPD13)	[57]	HD
RPH2	Q387R	Impaired trimer formation, decreased mtDNA levels, defective RNA import into mitochondria, and accumulation of mitochondrial dsRNA	Early-onset encephalomyopathy	[10,58]	RPH2
	R445E,R446E	Reduced degradation and enhanced polymerisation		[13]	
	S484A	Inhibited degradation and inhibited polyadenylation		[13]	
	P467S	Reduced flexibility of S1 domain, impaired RNA binding and degradation, and insufficient nuclear RNA import into mitochondria	Early-onset encephalopathy	[15,59]	
	E475G	Defective trimer formation, reduced ssRNA binding and degrading activity	Hereditary hearing loss	[10]	
	M485V		Developmental delay, defective brain development, and mobility and hearing impairment	[60]	
	G499R	Reduced flexibility of S1 domain and impaired RNA binding and degradation	Hearing loss, hypotonia, defective eye movements, and neuropathy leukodystrophy	[15,39,61]	
	A507S	Defective mtRNA accumulation, splicing defects, and impaired activity and levels of mitochondrial OXPHOS complexes.	Seizures, microcephaly, developmental eye defects, and hearing impairment	[62]	
	V509I		Hypoglycaemia, diabetes mellitus, hearing impairment, encephalopathy, and hepatic fibrosis	[63]	
	A510P	Impaired mitochondrial translation, impaired hPNPase assembly, and decreased hPNPase levels in blood cells	OXPHOS deficiency	[56]	
	T531R	Defective mtRNA accumulation, splicing defects, and impaired activity and levels of mitochondrial OXPHOS complexes		[62]	
	D538a, D544G	Reduced degradation and enhanced polymerisation		[13]	
KH	A684T		Hearing impairment paralysis of eye muscles and OXPHOS deficiencies accumulation of unprocessed mtRNA	[62]	KH
	Q672RfsX18		Spinocerebellar ataxia	[64]	
S1	K697NfsX6		Spinocerebellar ataxia	[64]	S1
	D713Y	Weak binding with Suv3, impaired RNA degradation, and reduced protein level	Combined oxidative phosphorylation deficiency, truncal hypotonia, nystagmus, and mitochondrial dsRNA accumulation	[15,39]	
	R715X (X = Termination)		Sensory ataxic neuropathy	[65]	
	M745T		Hearing impairment, neurodegeneration, and ataxia	[66]	

## hPNPase has been linked to apoptosis

Apoptosis is a tightly regulated form of programmed cell death that eliminates unwanted or damaged cells without triggering an inflammatory response. This essential physiological process plays a key role in tissue remodelling during development, the removal of damaged or dysfunctional cells, immune defence against pathogens and cancer surveillance. Mitochondria serve as a central hub for orchestrating apoptotic signalling, and hPNPase has been implicated in this process by facilitating the apoptotic RNA decay, highlighting its role in the regulation of cell death.

hPNPase is thought to induce apoptosis via multiple pathways. One well-characterised mechanism involves mitochondrial outer membrane permeabilisation, which results in the release of hPNPase into the cytoplasm, where it degrades polyadenylated RNAs [4,26]. Experimental evidence shows that overexpression of hPNPase, or its artificial localisation to the cytoplasm, enhances apoptotic processes. Elevated hPNPase levels trigger a cascade of signals and events typical for senescent cells, such as down-regulation of a key transcription factor that regulates cell proliferation c-Myc, up-regulation of Mad1, decreased telomerase activity and G1 cell cycle arrest, ultimately leading to apoptosis [37]. In melanoma cells, hPNPase has been shown to contribute to interferon-β-mediated suppression of c-Myc mRNA, linking it to the regulation of oncogenic signalling pathways [68]. As c-Myc is a key transcription factor driving cell proliferation, these findings suggest a broader role for hPNPase in modulating cell fate decisions in cancer.

An alternative apoptotic mechanism involves activation of the dsRNA-dependent protein kinase (PKR) by hPNPase. PKR activation triggers the phosphorylation of eukaryotic initiation factor-2α (eIF2 α), which in turn activates the growth arrest and DNA damage-inducible gene GADD153. This cascade leads to the termination of protein synthesis and promotes apoptosis. Additionally, hPNPase-induced PKR activation contributes to the down-regulation of the antiapoptotic protein Bcl-xL, further tipping the balance towards cell death [69].

## Emerging roles of hPNPase

Several emerging roles of hPNPase position it as an enzyme broadly involved in inflammatory processes and cellular stress responses. hPNPase plays a critical role in maintaining mitochondrial homeostasis, primarily through the regulation of mtRNA degradation, a process that directly influences subsequent steps of gene expression within mitochondria. Consequently, silencing of the *PNPT1* gene disrupts this tightly controlled balance, leading to mitochondrial dysfunction. Such disturbances can trigger a cascade of events that compromise mitochondrial biology, ultimately promoting inflammation and resulting in the activation of stress signalling pathways. One notable example is hPNPase's role in the activation of the NLRP3 inflammasome in macrophages. The NLRP3 (NOD-like receptor family pyrin domain-containing 3) inflammasome is a large cytosolic multiprotein complex that assembles in response to a wide range of microbial motifs and danger signals, such as ROS and mt-dsRNA. These signals, often produced by stressed mitochondria, can trigger inflammatory responses. Importantly, the NLRP3 inflammasome assembly is scaffolded on the mitochondrial antiviral-signalling protein (MAVS), which also mediates type I interferon response to mt-dsRNA, known to accumulate in the absence of hPNPase [39]. Depletion of hPNPase in macrophages has been shown to activate the NLRP3 inflammasome, resulting in the release of interleukin-1β, a key pro-inflammatory cytokine [70].

Recent studies have uncovered a novel role for hPNPase in the context of viral infection. Some viruses, including adenovirus, murine cytomegalovirus and murine hepatovirus, suppress the integrated stress response, a host defence mechanism, by up-regulation of hPNPase expression. Notably, depletion of hPNPase significantly reduces the ability of these viruses to propagate [71]. This antiviral effect appears to be linked to the release of mt-dsRNA into the cytoplasm in hPNPase-depleted cells. The presence of mitochondrial mt-dsRNA in the cytosol triggers a cascade of signalling events, ultimately leading to translational arrest and inhibited viral replication.

Beyond inflammation and infection, hPNPase has been implicated in cancer development. Elevated hPNPase levels have been reported in liver cancer, particularly in primary tumours of hepatocellular carcinoma patients, where high expression correlates with poor clinical outcomes, suggesting its potential as a prognostic biomarker [72,73]. Transcription factors SP1 and NFY, both associated with poor cancer prognoses, have been shown to regulate *PNPT1* expression [73]. Furthermore, the oncoprotein TCL1 may interfere with hPNPase mitochondrial localisation by sequestering it in the cytoplasm, promoting a metabolic shift from oxidative phosphorylation to glycolysis – a hallmark of cancer and stem cell proliferation [18,74]. hPNPase activity is also modulated by epidermal growth factor receptor signalling. Specifically, EGFR-mediated phosphorylation of hPNPase at serine 776 blocks its activity, leading to c-Myc mRNA accumulation and contributing to radioresistance in breast cancer cells [75]. This raises questions about hPNPase localisation and regulation in cancerous conditions. Intriguingly, hPNPase has also been identified as a tumour-associated antigen in CD40-activated leukemic cells [76], further supporting its potential as a therapeutic target or biomarker in oncology.

Beyond its canonical RNA processing roles, hPNPase appears to regulate a wide range of cellular pathways. Transcriptomic analyses have shown that hPNPase directly and indirectly influences various genes involved in, for example, cholesterol biosynthesis, cell cycle regulation and chromosomal organisation [77]. Knockdown of *PNPT1* disrupted mitochondrial respiratory complexes and down-regulated accessory factors, including the mitochondrial anion transporter UCP2, contributing to mitochondrial dysfunction and broader cellular dysregulation [77].

## hPNPase was shown to aid in trafficking of RNA across mitochondrial membranes

One of the most debated and least understood functions of hPNPase is its role in RNA trafficking, a concept that remains controversial despite decades of research. If such a mechanism exists, it might be analogous to the activity of *Escherichia coli* PNPase, which forms an RNA carrier complex with the RNA chaperone Hfq [33]. However, direct evidence supporting a similar role for hPNPase in human mitochondria is still lacking.

Several noncoding RNAs have been reported to be imported into human mitochondria, both *in vivo* and *in vitro*, including tRNAs, 5S rRNA, H1 (the RNA component of RNase P), RMRP (the RNA component of RNase MRP), SAMMSON (a long non-coding RNA), TERC (the RNA component of telomerase) and several miRNAs [38,78–88]. hPNPase has been implicated in the mitochondrial import of several of these RNAs, in a manner independent of its catalytic activity [38,86]. Notably, recent studies have demonstrated that long noncoding RNAs (lncRNAs) constitute a significant portion of hPNPase-associated transcripts [89]. A common feature among many imported RNAs is the presence of a stem-loop structure of approximately 20 nucleotides, which has been proposed as a potential RNA recognition motif required for hPNPase-mediated transport from the cytoplasm to the mitochondrial matrix [38]. However, subsequent analyses indicate that PNPase recognises more complex RNA features rather than generic stem-loops. These include a preference for lower GC content, a higher unpaired nucleotide frequency and specific sequence motifs contributing to increased binding affinity [89]. Importantly, RNA trafficking between mitochondria and the nucleus may be bidirectional. hPNPase has recently been implicated in the retrograde transport of specific RNAs out of mitochondria, adding a new dimension to its proposed role in RNA transport [86,90].

Despite these findings, the molecular mechanisms underlying the selectivity of mtRNA import remain poorly understood. Recent research has identified adenine nucleotide translocase 2 (ANT2) as capable of transporting RNA across the mitochondrial inner membrane, suggesting that it may serve as a potential component of the mtRNA shuttling pathway [91] Nevertheless, no consensus mitochondrial-targeting RNA sequence or specific cytosolic proteins involved in RNA trafficking have been identified as of yet. Further adding to the controversy, some studies have questioned the biological relevance of certain nucleolar RNAs found in the mitochondrial matrix, proposing that their presence might result from experimental artifacts rather than genuine biological processes [92–94]. For example, in HeLa cells, the amount of MRP RNA detected in highly purified mitochondria was found to be negligible [92]; however, *in situ* hybridisation using probes against MRP RNA in fixed mouse heart sections revealed a clear mitochondrial signal, indicating its potential mitochondrial localisation in certain tissues [95]. Additionally, even the low levels of MRP RNA detected in mitochondria might still fulfil the enzymatic requirements of RNase MRP [96].

A similar controversy exists regarding the RNA component of RNase P, which has been shown to be imported into mitochondria by hPNPase [38,97,98]. However, the human mtRNase P has been shown to function without RNA, suggesting a protein-only mechanism [99,100]. The case of 5S rRNA is equally complex. While some evidence supports its PNPase-dependent presence in mitochondria [38,80,81,101,102], structural models of the mitochondrial ribosome consistently depict a 5S rRNA-free architecture [103–106]. These concerns highlight the need for more rigorous experimental validation and standardised methods to confirm the presence and functional relevance of ncRNAs within mitochondria.

Cytoplasmic tRNA molecules were also shown to be imported into human mitochondria. Although humans encode a full set of 22 tRNAs within their mitochondrial genome, unlike other organisms that depend on cytoplasmic tRNA import (reviewed in [81]), directional deep sequencing of the human mitochondrial transcriptome across multiple cell lines and tissues revealed the presence of small amounts of cytoplasmic tRNAs, where they appeared in a processed, mature form [79]. The same study confirmed the presence of low levels of other noncoding RNAs, including 5S rRNA, MRP RNA and RNase P RNA, within isolated mitoplasts, indicating their potential involvement in mitochondrial function [79]. Finally, several microRNAs (miRNAs) have been found within mitochondria, with some studies suggesting the involvement of hPNPase in their import. For instance, miRNA-378, which has been implicated in the regulation of bioenergetics in type 2 diabetes mellitus, was shown to require hPNPase for its mitochondrial localisation [107].

## Discussion

**Figure 2:**
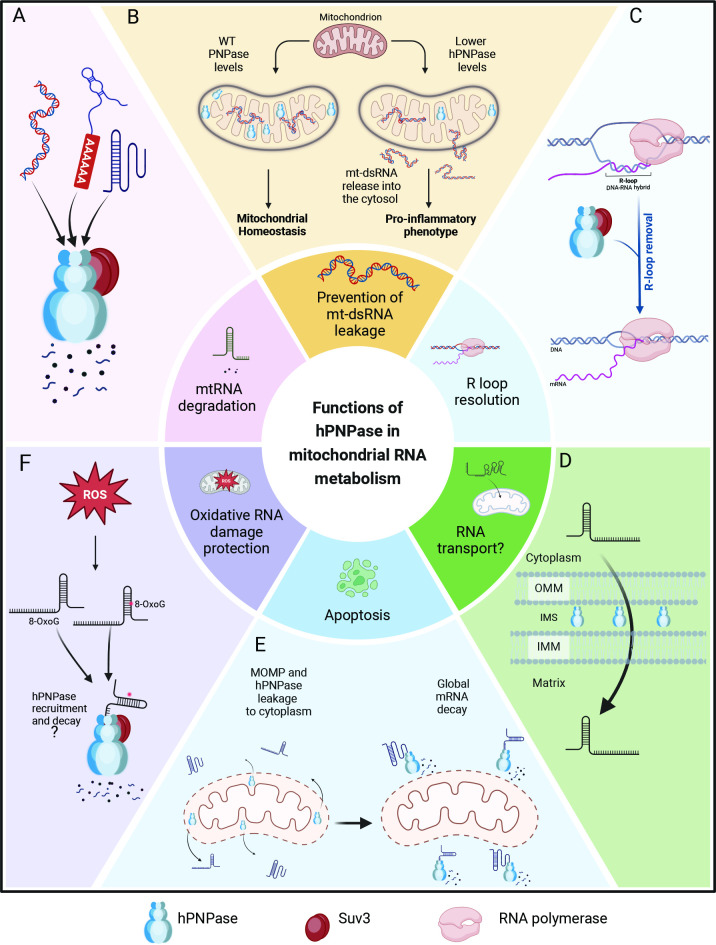
Functions of hPNPase in human mitochondria. (**A**) In conjunction with the helicase Suv3, hPNPase drives the degradation of multiple mitochondrial RNA species, forming the mitochondrial degradosome complex. (**B**) hPNPase contributes to mitochondrial homeostasis by preventing the accumulation and release of mt-dsRNA. A decrease in hPNPase levels leads to the escape of mt-dsRNA into the cytoplasm, triggering inflammation. (**C**) The mitochondrial degradosome, comprising hPNPase and Suv3, prevents the accumulation of R-loops, which are detrimental DNA-RNA hybrids that can disrupt replication and transcription. (**D**) hPNPase is proposed to mediate RNA transport from the cytosol to mitochondria, facilitating the delivery of RNA molecules. (**E**) During apoptosis, hPNPase is released into the cytoplasm following mitochondrial outer membrane permeabilisation (MOMP), where it mediates global RNA decay. (**F**) hPNPase is involved in the clearance of oxidatively damaged RNA, protecting mitochondrial RNA integrity. hPNPase, polynucleotide phosphorylase; mt-dsRNA, mitochondrial double-stranded RNA. Created in BioRender. Bandyra, K. (2025) https://BioRender.com/z60l592

Despite decades of research, hPNPase remains a puzzling enzyme, implicated in a wide range of cellular processes (Figure 2). Conflicting reports regarding its mitochondrial roles, along with an incomplete understanding of its multifaceted functions, make it challenging to form a cohesive picture of this evolutionarily conserved protein. A significant source of these discrepancies likely arises from the diverse experimental systems and methodologies used to study hPNPase. Notably, variations in silencing strategies, cell lines and analytical methods have produced conflicting results, even with respect to hPNPase’s most basic and well-accepted function: RNA degradation. While some studies report a significant impact of hPNPase on mtRNA stability and steady-state levels [6,35], others have found no such effect [36], highlighting the variability and complexity of interpreting hPNPase function across different experimental systems. Similarly, its role in mitochondrial translation is debated, with evidence suggesting hPNPase as both affecting mitochondrial protein synthesis [38] and lacking an effect on mitochondrial protein levels [35,36].

These inconsistencies extend to its proposed function in mtRNA transport and its puzzling localisation to the IMS. Although hPNPase is undeniably a key player in mitochondrial biology, many aspects of its molecular function remain elusive. Key questions persist: What is the specific role of hPNPase within the IMS? Does its presence there primarily serve as a reservoir for release during apoptosis, or does it have additional yet-undiscovered roles? How does hPNPase distinguish between normal mtRNAs and those marked for degradation? Is it involved in the processing of mitochondrial non-coding RNAs? Does it play a role in RNA-mediated mitochondria-nucleus cross-talk? Furthermore, could the function of hPNPase vary across tissues with high energy demands, such as the brain or heart, reflecting tissue-specific adaptations or regulatory mechanisms? These open questions highlight the need for further research to elucidate the precise mechanisms by which hPNPase contributes to mtRNA metabolism and broader mitochondrial function.

## Open Access

This article has been published open access under our Subscribe to Open programme, made possible through the support of our subscribing institutions, learn more here: https://portlandpress.com/pages/open_access_options_and_prices#conditional

## Abbreviations

MTS: mitochondrial targeting sequence
PNPase: polynucleotide phosphorylase
hPNPase: human PNPase
mt-dsRNA: mitochondrial double-stranded RNA
